# Diphtheritic Hemiplegia

**Published:** 1906-01-13

**Authors:** 


### DIPHTHERITIC HEMIPLEGIA.

Dr. J. D. Rolleston [1] reports a case of this rare condition. The patient, a boy of six years, was admitted to hospital with faucial diphtheria on November 21, 1904. The severe nature of the case was illustrated by such events as weak and irregular action of the heart, considerable enlargement of the liver and very scanty urine. On December 8 there was observed paralysis of right lower face and of right upper limb, with also some palsy of the right lower limb. There was at the same time motor aphasia. Both knee-jerks were absent, and the plantar response on the right side was of the extensor type, that of the left being normal. On December 10 there was paralysis of accommodation, and on the 23rd paralysis of the muscles of deglutition. Signs of improvement were noted early in the following month, and when the patient left the hospital at the end of March his speech had returned, and he could walk fairly well, though there was still some weakness of the right grasp.

Hemiplegia following diphtheria is a very unusual condition. It differs from the ordinary forms of diphtheritic paralysis in that it is dependent, not on a primary lesion of the nervous apparatus, but on a vascular event—thrombosis, embolism, or hæmorrhagic encephalitis. In all the reported cases the initial faucial attack was severe. Complete recovery is rare; such consequences as contracture, hemichorea, athetosis, and idiocy have been noted.

[1] Med. Press and Circ., Dec. 20, 1905.

